# Generation and characterization of Kctd15 mutations in zebrafish

**DOI:** 10.1371/journal.pone.0189162

**Published:** 2017-12-07

**Authors:** Alison Heffer, Gregory D. Marquart, Allisan Aquilina-Beck, Nabil Saleem, Harold A. Burgess, Igor B. Dawid

**Affiliations:** Division of Developmental Biology, *Eunice Kennedy Shriver* National Institute of Child Health and Human Development, National Institutes of Health, Bethesda, MD, United States of America; Texas A&M University, UNITED STATES

## Abstract

Potassium channel tetramerization domain containing 15 (Kctd15) was previously found to have a role in early neural crest (NC) patterning, specifically delimiting the region where NC markers are expressed via repression of transcription factor AP-2a and inhibition of Wnt signaling. We used transcription activator-like effector nucleases (TALENs) to generate null mutations in zebrafish *kctd15a* and *kctd15*b paralogs to study the in vivo role of Kctd15. We found that while deletions producing frame-shift mutations in each paralog showed no apparent phenotype, *kctd15a/b* double mutant zebrafish are smaller in size and show several phenotypes including some affecting the NC, such as expansion of the early NC domain, increased pigmentation, and craniofacial defects. Both melanophore and xanthophore pigment cell numbers and early markers are up-regulated in the double mutants. While we find no embryonic craniofacial defects, adult mutants have a deformed maxillary segment and missing barbels. By confocal imaging of mutant larval brains we found that the torus lateralis (TLa), a region implicated in gustatory networks in other fish, is absent. Ablation of this brain tissue in wild type larvae mimics some aspects of the mutant growth phenotype. Thus *kctd15* mutants show deficits in the development of both neural crest derivatives, and specific regions within the central nervous system, leading to a strong reduction in normal growth rates.

## Introduction

The family of potassium channel tetramerization domain (KCTD) proteins has diverse biological functions, including protein degradation, DNA replication, regulating the hedgehog pathway, and transcriptional repression [1]. While these proteins all share a BTB/POZ (BR-C, ttk and bab/Pox virus and Zinc finger) protein-protein interaction domain near the N-terminus, they are structurally very different outside of this region [1–4]. Mutations or variants in several KCTD members have been implicated in various human diseases, including cancer, neurological diseases and metabolic disorders (reviewed in [5]), providing medical significance for the further study of this family of proteins.

In zebrafish, we previously reported that Kctd15 has a function during embryogenesis in the neural crest (NC) [6, 7]. The NC is a population of cells unique to vertebrates whose derivatives migrate and differentiate into a variety of cell types throughout the body, including craniofacial bones, pigment cells and much of the peripheral nervous system [8, 9]. Kctd15 is expressed first in the neural plate border region adjacent to the NC, and has a role in defining the NC region by repressing transcription factor AP-2 activity and inhibiting Wnt signaling [6, 7].

Kctd15 function has also been examined in other organisms. In the fruit fly *Drosophila*, Kctd15 is involved in both male aggression [10] and feeding frequency, as loss of the Kctd15 ortholog Twz in octopaminergic neurons resulted in flies consuming more food [11]. Genome wide association studies (GWAS) in humans have found significant linkage between *KCTD15* and obesity [12–15]. Additionally, *kctd15* gene expression levels in the hypothalamus of chickens and mice are related to diet, further supporting a role of Kctd15 in obesity [16, 17].

Here, we report the generation and characterization of zebrafish Kctd15 mutants. Zebrafish have two *kctd15* paralogs (*kctd15a* and *kctd15b*) that have overlapping expression domains in the early NC, but then diverge as embryogenesis progresses [6, 18]. We find that homozygous mutants in either paralog alone show no phenotype, but *kctd15a/b* double mutants, while viable and fertile, are smaller in size and exhibit abnormalities in several NC-derived tissues including pigment cells and craniofacial bones. These mutants are lacking a fully formed maxilla bone in the upper jaw of adult fish, as well as the sensory barbels normally present on this craniofacial segment. Additionally, these mutants are missing the torus lateralis (TLa), a midbrain region which is implicated in gustatory networks in other fish species [19–22]. Ablation of this region resulted in a significant growth delay in mutant larvae compared to controls, though *kctd15* mutant larvae were still smaller. Together, these results suggest that it is a combination of the deformed maxillary bone and sensory problems that lead to the smaller size of the mutants.

## Results

### Generation of zebrafish kctd15 mutants

Zebrafish have two *kctd15* paralogs, *kctd15a* and *kctd15b*, which are 91% identical and 98% similar at the amino acid level, with complete identity in the protein-protein interacting BTB domain. We used transcription activator-like effector nucleases (TALENs; [23]) to generate null mutations in each *kctd15* paralog for further characterization and functional studies (S1 Fig). Several mutations were recovered, with ~30% fish carrying at least one mutation. For further characterization, we chose *kctd15aΔ23*, carrying a 23 base-pair (bp) deletion upstream of the BTB-encoding sequence in exon 2, and *kctd15bΔ19* carrying a 19 bp deletion at the beginning of the BTB-containing region in the third exon. Both deletions lead to frame-shifts and introduced premature stop codons (S1A and S1B Fig). While the *kctd15* paralogs have overlapping expression domains in early development and therefore might compensate for each other, divergent expression patterns after somitogenesis [6] suggested possible subfunctionalization later in development. However, the *kctd15aΔ23* and *kctd15bΔ19* single maternal-zygotic homozygous mutants show no visible phenotype and are viable and fertile.

We crossed the two single mutant lines to generate a *kctd15a/b* double mutant (hereafter referred to as *kctd15* mutant). Whereas embryos injected with Kctd15a/b morpholinos did not survive past 5 dpf [6], null double mutants do survive to adulthood but show a developmental delay. *Kctd15* mutant fish become fertile at 4–6 months, almost twice the age of wild-type siblings. Since these mutants survive past embryogenesis, we were able to study late-onset phenotypes, as discussed below.

We believe that the mutations introduced by TALENS and described here are null mutations. No Kctd15 antibody amenable to immunohistochemistry in zebrafish is available, so we checked for mutant Kctd15 protein expression by other means (S1C and S1D Fig). We cloned wild-type and mutant *kctd15a* and *kctd15b* sequences from embryonic cDNA into expression vectors that supply an N-terminal Flag tag and expressed the constructs in cell culture. Using a Kctd15 antibody against a C-terminal epitope that is suitable for immunoblotting, and anti-Flag antibody, we observed the expected ~29–30 kDa proteins by expressing wild type constructs, but no detectable protein with mutant constructs (S1C Fig). Further, we analyzed *kctd15a* and *kctd15b* transcript levels in our double mutants. Kctd15 has previously been reported to repress its own transcription [24]. Both *kctd15a* and *kctd15b* transcript levels are significantly up-regulated at several developmental time points in *kctd15* mutants (S1D Fig). This suggests that transcription of both *kctd15* paralogs are “de-repressed” by the absence of functional Kctd15 protein, supporting the conclusion that our mutants are functional null.

### kctd15 mutants exhibit phenotypes associated with up-regulation of NC cells

#### I. Slight expansion and up-regulation of several NC cell markers

**Fig 1:**
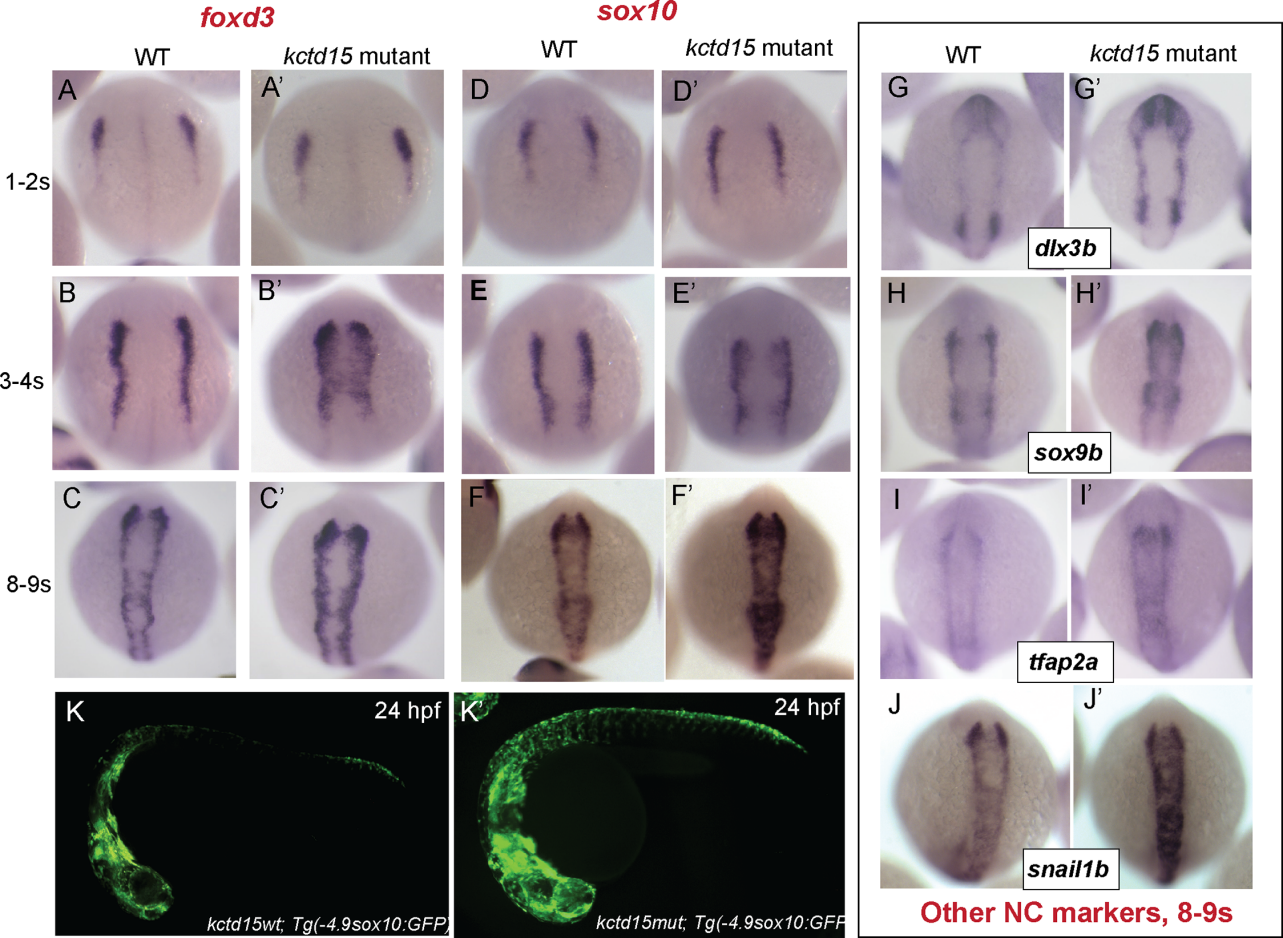
*kctd15* mutants show up-regulation of several NC gene markers. *foxd3* (A-C) and *sox10* (D-F) expression is indistinguishable between WT and mutants very early in NC development, but by 3-4s, expression of these markers shows both an expansion and up-regulation in expression, which persists at 8-9s. Other NC markers, including *dlx3b* (G), *sox9b* (H), *tfap2a* (I) and *snail1b* (J) also show up-regulation and expansion in our mutants at 8-9s. Additionally, an increase in NC cell number is apparent at 24hpf in *kctd15* mutants, as visualized in a *sox10-*GFP reporter construct (K).

In zebrafish, the population of cells destined to become the NC localize to the neural plate border (NPB) at ~10.5 hpf, later delaminate and migrate to multiple locations where they differentiate to pigment cells, the craniofacial cartilage and bones, much of the peripheral nervous system, and other cell types (reviewed in [8, 9, 25]). The transcriptional regulatory networks underlying NC development have been well-characterized (reviewed in [26–28]). Previously we showed that Kctd15 overexpression inhibits NC formation as visualized by changes in gene marker expression patterns. We suggested that the role of Kctd15 was to delimit the early NC domain [6]. At a molecular level this effect on NC development is likely due to inhibition of Wnt signaling and of the function of transcription factor AP-2 [6, 7, 29, 30]; Wnt signaling and AP-2 function are essential for NC development [31, 32]. To further study the role of Kctd15 in NC formation, we examined expression of several NC markers in *kctd15* mutants (Fig 1). The expression patterns of the early NC markers *foxd3* and *sox10* were unchanged in Kctd15 mutants at 1–2 somites (Fig 1A, 1A’, 1D and 1D’), but showed minor expansion at 3–4 somites (Fig 1B, 1B’, 1E and 1E’), which persists at least to 8–9 somites (Fig 1C, 1C’, 1F and 1F’). Additional markers, *dlx3b*, *sox9b*, *tfap2a*, and *snail1b* were examined at 8–9 somite stages, when all gave evidence of a modest level of expansion and up-regulation of expression (Fig 1G–1J’). This expansion of the NC domain suggests that *kctd15* mutants contain more NC cells, and therefore are expected to generate an excess of NC derivatives. To test this, we crossed our *kctd15* mutant into fish that contain a transgenic sox10-GFP reporter construct that labels most migrating NC cells [33]. An increase in GFP expression region and intensity was observed at 24 hpf in mutants compared to wild-type, supporting the view that the NC domain is expanded in *kctd15* mutant embryos (Fig 1K and 1K’).

#### II. *kctd15* mutants have increased pigmentation

**Fig 2:**
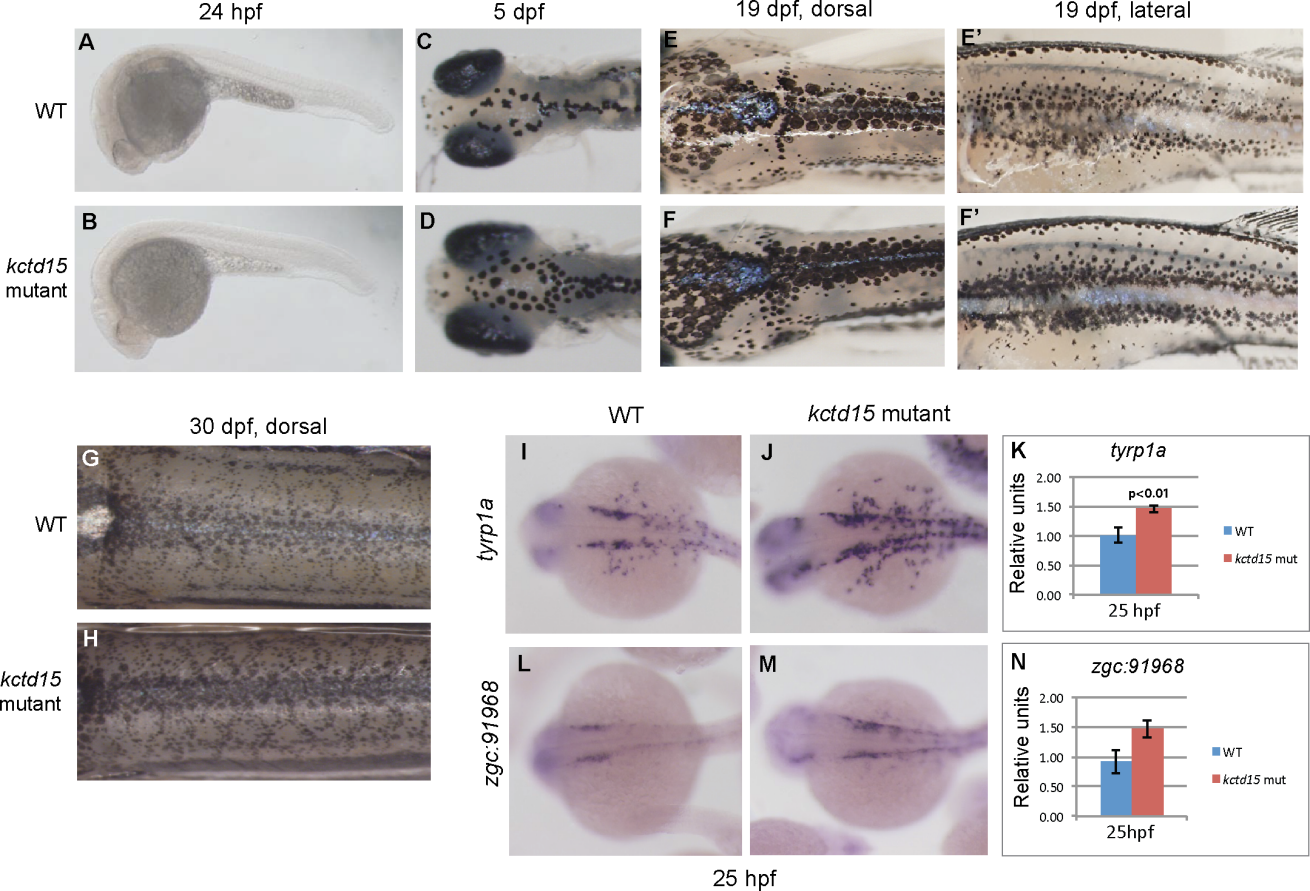
*kctd15* mutants have more melanophores. While there is no premature appearance of melanophores in our mutants (A,B), by 5 dpf there are visibly more on the dorsal side of the head (C,D), and by 19 dpf, there is a marked increase in melanophore pigment cells both on the dorsal and lateral sides of the larvae (E,F). This increase in pigmentation is still seen at 30 dpf (G,H). Examination of transcript levels and expression patterns in 25 hpf embryos revealed an up-regulation of the early melanophore markers *tyrp1a* (I-K) and *zgc*:*91968* (L-N).

Pigment cell precursors that originate from the NC [34, 35] can be detected as early as ~24 hpf in zebrafish. These cells later differentiate into the three types of pigment cells found in adults—melanophores, xanthophores and iridophores. To check whether the apparent increase in the NC results in an increase in pigmentation we compared pigmentation patterns in wild type and *kctd15* mutant embryos and larvae. No premature appearance of melanophores was seen by 24 hpf (Fig 2A and 2B) and no difference was apparent in melanophore pigmentation patterns at 48 hpf. However, sometimes at 3 dpf and always by 5 dpf, there are visibly more melanophores in the mutants (Fig 2C and 2D), a phenotype that is maintained through larval stages at 19 dpf (Fig 2E and 2F) and 30 dpf (Fig 2G and 2H). The observed increase in melanophores by 5 dpf suggests a possible up-regulation of genes involved in early melanophore development. Therefore, we examined expression levels of several genes involved in early melanophore development by *in situ* hybridization and quantitative PCR (qPCR; Fig 2I–2N, S2 Fig). We find up-regulation of *typr1a* [36] and another gene expressed in migrating NC cells destined to become melanophores, *zgc*:*91968* ([37]; Fig 2I–2N). Somewhat surprisingly, we find that the “master regulator” of melanophore development, *mitfa* [38, 39], was not up-regulated at 25 hpf in the *kctd15* mutants (S2A–S2C Fig), but was significantly up-regulated at 48 hpf (S2C Fig); similar results are seen with *dct* ([34, 40]; S2D Fig). Other genes known to be involved in melanophore development, *kita* [41], and *tyr* [42], were not significantly up-regulated at 25 hpf or 2 dpf (S2D and S2E Fig).

**Fig 3:**
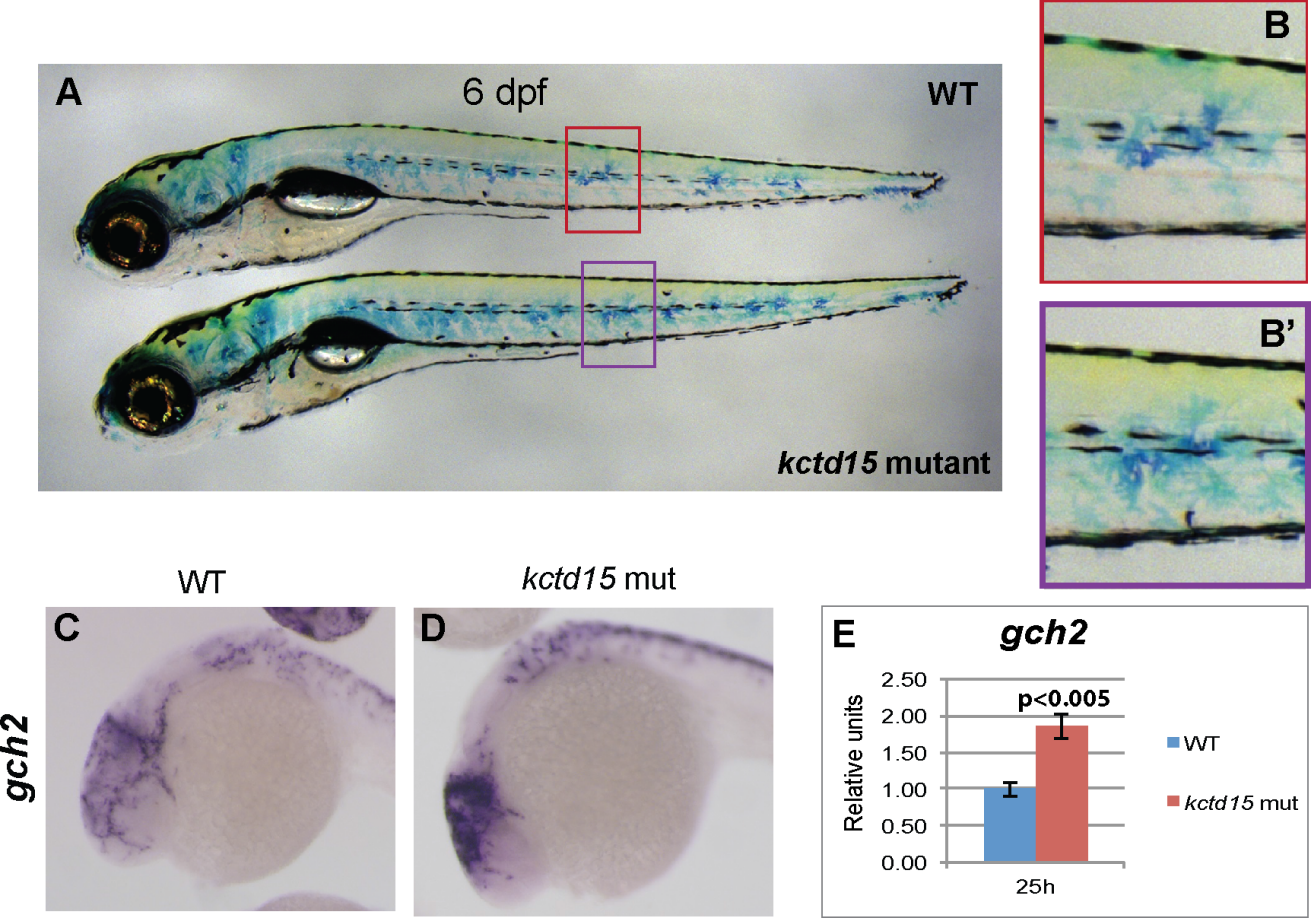
*Kctd15* mutant larvae have more xanthophores. At 6 dpf, there are more mature xanthophores, as indicated by methylene blue staining (A); however, their appearance is just like WT (B). Examination of genes involved in xanthophore specification showed that there was an increase in abundance of *gch2* transcripts at 25 hpf (C-E), most notably in the cranial pigment cells.

Xanthophore number, as visualized by immunostaining with Pax3/7 antibody, did not differ significantly between wild type and mutants at 30 hpf (S3A and S3B Fig; [43, 44]). However, we see an apparent increase in xanthophore number at 6 dpf by methylene blue staining (Fig 3A and 3B), while the appearance of these cells in mutant larvae is normal (Fig 3B and 3B’). There is also a more “gold” appearance on the dorsal side of mutant embryos, indicating a larger number of mature xanthophores. We examined the expression of several genes involved in early xanthophore development, including *gch2* [45], *xdh* [46], *csf1ra* [47] and *pax7b* [48, 49] by ISH and qPCR (Fig 3C–3E; S3C–S3E Fig). Only *gch2* is significantly up-regulated at 25 hpf when xanthophore development begins (Fig 3); the increase is seen primarily in the head region of the mutant embryos (Fig 3C and 3D).

Finally, we inquired whether iridiphores are increased in the mutants by measuring expression levels of iridophore markers *ednrab* [50], *pnp4a* [44], *ltk* [51], and *tfec* [52] (S4 Fig). We found no significant change in expression of these markers at 25 or 48 hpf, suggesting that iridophore development is unaffected in *kctd15* mutants at this time. Due to the increased number of melanophores, we could not quantify mature iridophores in the mutant larvae. Taken together, we find that in zebrafish, Kctd15 regulates NC derivatives destined to become melanophores and xanthophores.

#### III. Mutations in *kctd15* do not affect glial cell formation

As reported above, we observe an increase in NC cell number and specific types of pigment cells. We wanted to know if other cell types derived from the NC were also affected, including glial cells of the developing peripheral nervous system [53, 54]. *In situ* hybridization of glial markers *foxd3* and *sox10* at 48 hpf showed no apparent change in number of cranial glial cells (S5A and S5B Fig) or trunk glial cells (S5C and S5D Fig) in our mutants compared to wild-type. These results suggest that loss of Kctd15 function does not result in a broad up-regulation of the NC, but is specific to certain cell populations.

#### IV. Kctd15 mutants exhibit craniofacial abnormalities as adults

**Fig 4:**
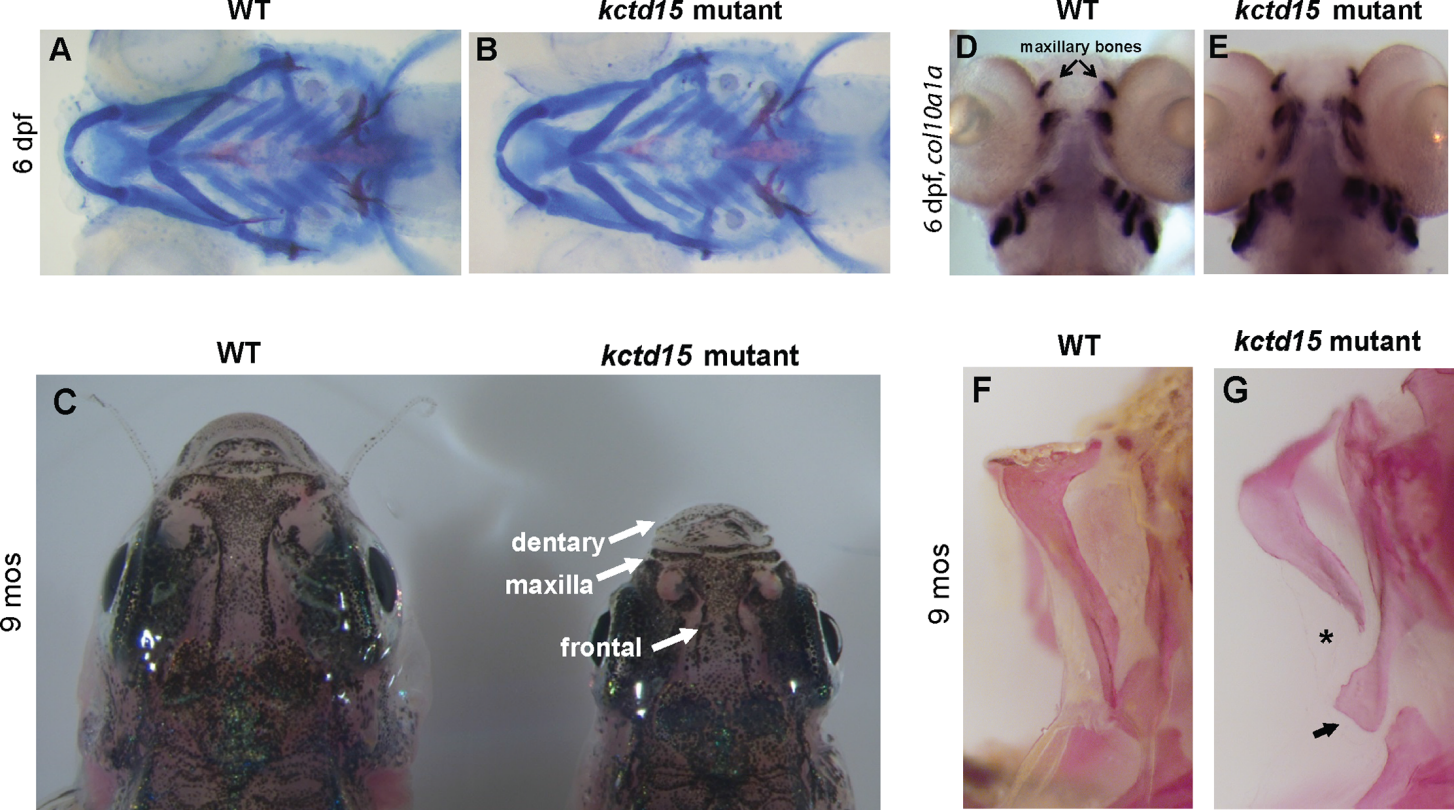
Kctd15 is required for proper jaw formation later in development. Alcian blue/Alizarin red staining does not reveal any early structural abnormalities in the patterning of *kctd15* mutant jaws (A,B). However, adult mutants exhibit shortening of several jaw elements, including the dentary, maxillary and frontal regions (C). While mRNA staining at 6 dpf for *col10a1a* showed no difference in patterning of early craniofacial bones (D,E), adult double mutants lack a properly formed maxilla bone (F,G; abnormalities are indicated by an asterisk and arrow).

Much of the craniofacial skeleton and musculature in vertebrates is derived from the NC [9, 55]. Since there is mis-regulation of NC cell number and derivatives in *kctd15* mutants, we checked for defects in the craniofacial skeleton [56]. Cranial cartilage and bone visualized by Alcian Blue and Alizarin Red staining, respectively, showed no abnormalities in mutant larvae compared to wild-type siblings (Fig 4A and 4B), but adult mutant fish exhibit a general “shortening” of the jaw and head elements, including the frontal and dental bones (Fig 4C), and are lacking a properly formed maxillary bone (Fig 4F and 4G). This malformation of the maxillary region likely is not due to an early mis-patterning of this region during embryogenesis, as staining with *col10a1a* mRNA, which is expressed in the developing craniofacial bones starting around 4–5 dpf [57], shows that the development of the maxillary bone is initiated in mutants and has comparable *col10a1a* expression to wild-type (Fig 4D and 4E). These results indicate that Kctd15 has a function in the formation of the maxilla at late larval stages.

**Fig 5:**
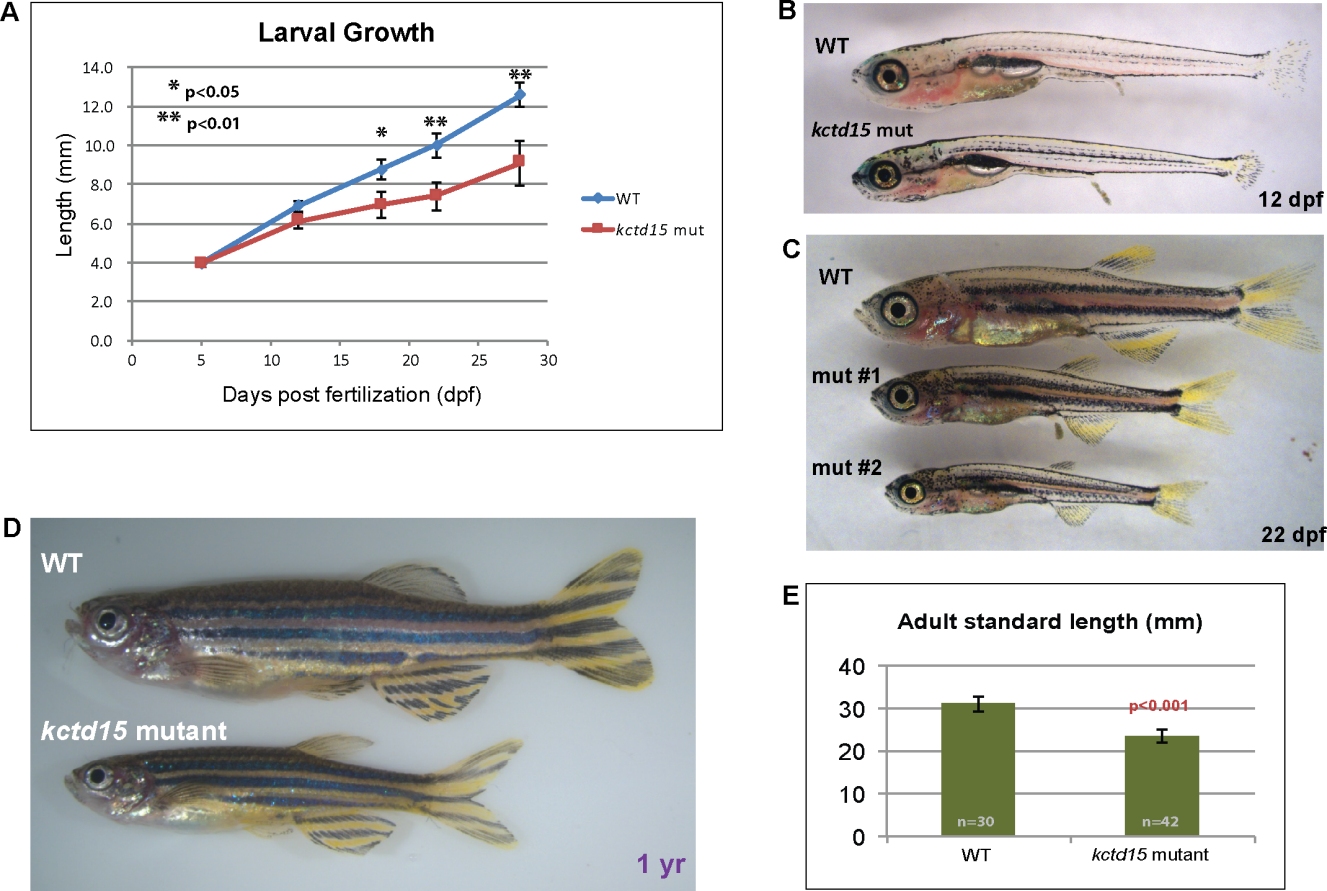
*Kctd15* mutants are smaller in size. A) Growth of wild-type and mutant larvae, measured at 5, 12, 18, 22 and 28 dpf. While the difference in size is noticeable at 12 dpf (B), the difference is not significant until 18 dpf (C), when a range of sizes is seen, ranging from somewhat smaller (mut #1) to much smaller (mut #2). Adult mutants remain significantly smaller than wild-type siblings, even after a year (D,E).

Interestingly, we also find that *kctd15* mutants are missing all facial barbels, the sensory organs found on the face of fish, which contain many of the taste buds (S6 Fig). This is likely not correlated with the smaller size of *kctd15* mutant fish, as barbels begin growing at standard length (SL) ~10–12 mm [58], and the adult mutant fish always surpass this length (Fig 5). Barbels may be missing in the mutants because the area where they normally develop on the maxillary segment isn’t properly formed (arrows, Fig 4G), and/or due to structural differences in the brain (see below).

### Kctd15 mutants show a slow growth/small size phenotype

While *kctd15* mutants are viable and fertile, they never reach the size of wild-type siblings (Fig 5) and take longer to reach sexual maturity. This size difference is not seen at 5 dpf, and while noticeable at 12 dpf (Fig 5B), is not significant until later in development (Fig 5A and 5C). Mutant larvae continue to remain smaller than wildtype siblings throughout larval development and into adulthood (Fig 5A, 5D and 5E). There are several possible explanations for the smaller size of the mutants, none of which are mutually exclusive. In addition to structural defects in the maxilla segment and missing barbels which might hinder proper feeding (Fig 4), additional possibilities include decreased growth hormone production [59], another brain abnormality that affects hormone production or distribution, or other mechanisms. We tested the level of growth hormone mRNA (*gh*) in *kctd15* mutants by qPCR and *in situ* hybridization at 48 hpf, when the hormone is detected in the developing pituitary gland [60], and found that while there was a general trend towards lower *gh* levels, this difference was not significant (S7 Fig). Interestingly, whereas wild-type larva showed a rosette-like expression pattern of *gh*, *kctd15* mutants had more variation in the number and organization of these cells (S7B Fig). However, while expression levels are not significantly lower, we have not excluded possible post-transcriptional effects on growth hormone levels in the *kctd15* mutants.

### Kctd15 is required for the formation of the torus lateralis in zebrafish

**Fig 6:**
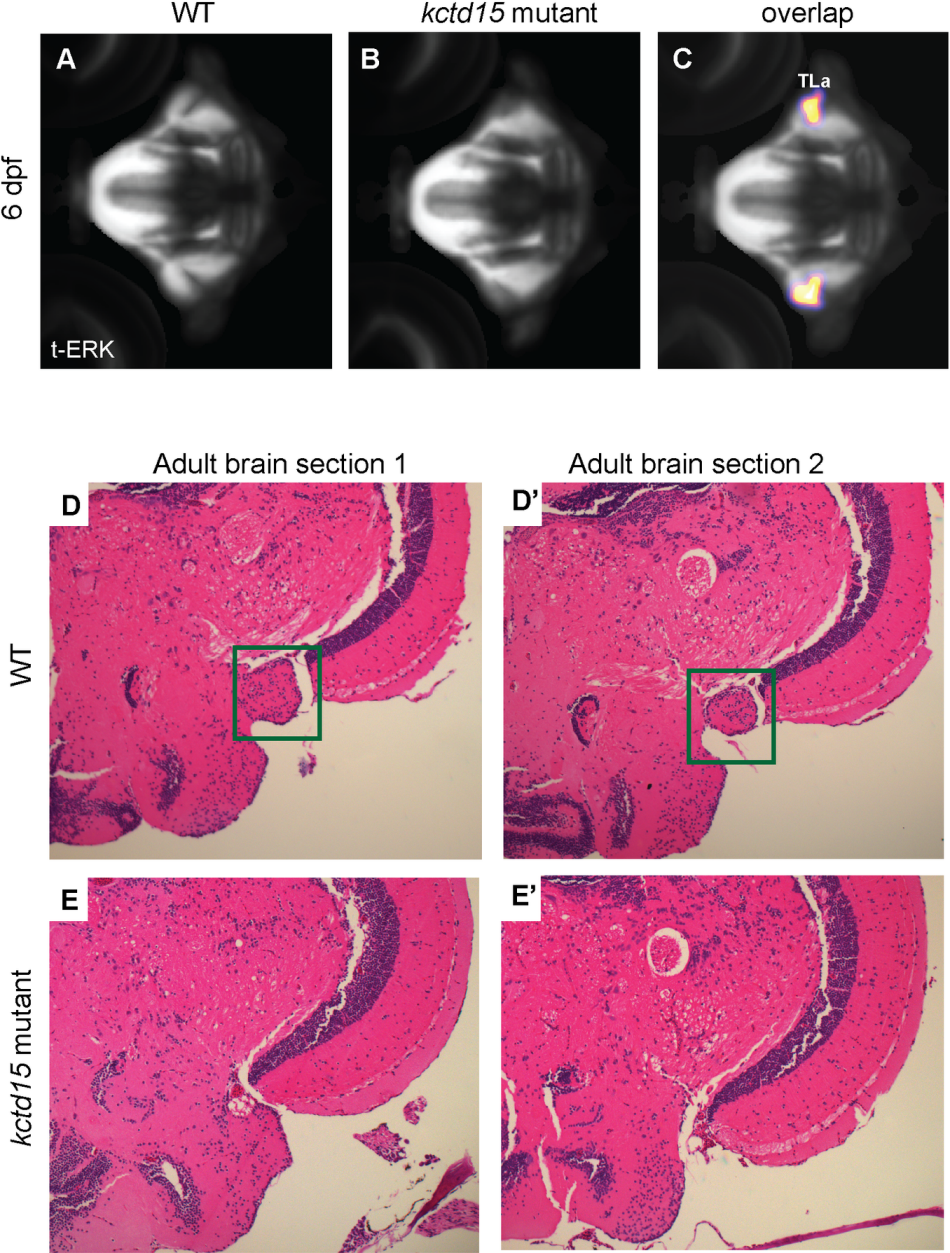
*Kctd15* mutants are missing the torus lateralis (TLa). Total-ERK (tERK) antibody staining of (A) wild-type and (B) mutant 6 dpf larvae. C) Pixels whose intensity values are statistically significantly different between wildtype and mutant brains (p < 0.05, N = 14 per group, see Methods for statistical comparison procedure). Sectioning and H+E staining of a large region of adult (5 month old) wild-type (D) and mutant (E) brains revealed that this structure (green square in D) is absent throughout development. At least fifteen larval brains and five adult brains from each genotype were analyzed to confirm these results.

While *kctd15* mutants lack gross morphological abnormalities early in development, it is possible more subtle structural defects exist. To search for subtle structural abnormalities in *kctd15* mutant brains, we labeled larvae using a tERK antibody, which preferentially labels brain structure, and looked for differences in confocal image stacks aligned to a common reference and subjected to brain-wide voxel-wise analysis. Based on segmentations from Z-Brain [61] aligned to the Zebrafish Brain Browser [62], a region in the mid-brain that mapped to the torus lateralis (TLa) was identified as missing in *kctd15* mutants (Fig 6A–6C). This absence is not due to a delay in growth or development of this region, as the TLa was missing in adult *kctd15* mutant brains as well (Fig 6D and 6E). The TLa has been implicated in sensory functions in other fish [19–22], so it is possible that mutant zebrafish are impaired in taste or smell, inhibiting feeding.

**Fig 7:**
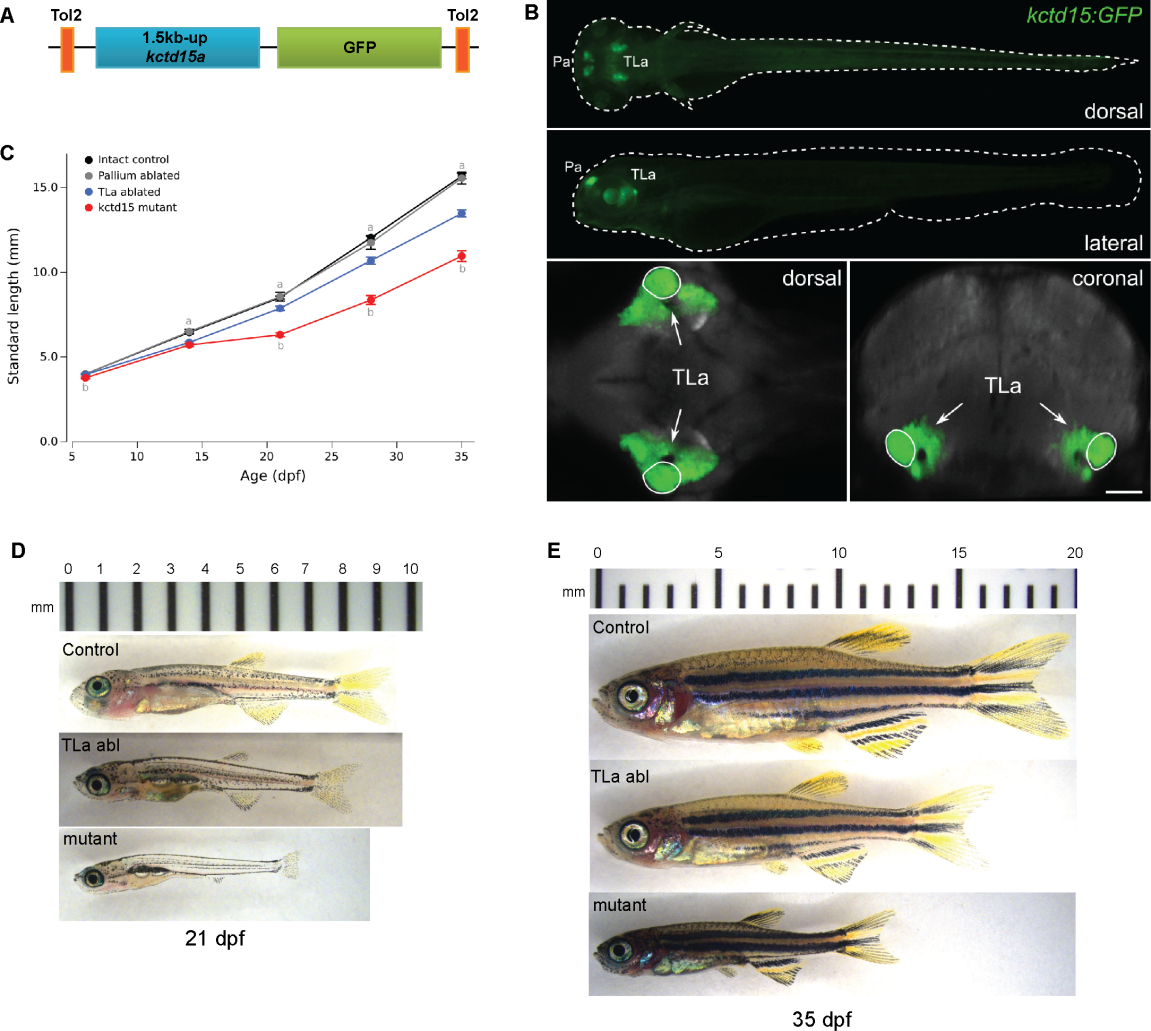
TLa ablation resulted in larva of smaller size. A) Construct design for generating a stable GFP transgenic line. 1.5kb upstream of the initiation codon of *kctd15a* was cloned upstream of GFP and inserted into the genome using Tol2. B) A dorsal maximum projection of an average representation of the transgenic line at 6 dpf (green) overlayed on vGLUT expression (gray) shows strong expression in the pallium region of the forebrain and torus lateralis in the midbrain. The superimposed heat map indicates the region missing in our *kctd15* mutants. C) Growth measurements taken over the course of 5 weeks of non-ablated control larva (see methods; n = 39–55 per time point), control pallium ablated (n = 8 per time point), TLa ablated (n = 24–26 per time point) and *kctd15* mutants (n = 28 per time point). At 6 dpf entire larval length was measured, at all other time points standard length (SL) was measured. See text for ANOVA results. For post-hoc t-tests: (a) indicates p < 0.05 for torus lateralis (TLa) ablated compared to intact control or pallium ablated controls. (b) indicates p < 0.05 for TLa compared to kctd15 mutants.

It is notable that the TLa produces growth hormone releasing hormone (GHRH) in the adult zebrafish brain [63]. GHRH functions by binding to the growth hormone releasing hormone receptor and stimulates the release of growth hormone [64]. We hypothesized that the smaller size of our *kctd15* mutants could be due, at least in part, to a decreased production of GHRH and consequent reduction of GH availability. To investigate whether the TLa has a role in zebrafish growth we ablated this region at 5 dpf and measured growth for the subsequent 5 weeks. We generated a stable transgenic line expressing GFP driven by a 1.5kb region upstream of the *kctd15a* gene (*Tg*(1.5kb *kctd15a*-GFP); Fig 7A), which we found to be expressed in a region of the larval brain that overlaps with the TLa (Fig 7B, S8 Fig). The GFP label served as guidance in locating the TLa. Ablation of the TLa region, but not a control region in the pallium, resulted in larva that were on average smaller in size (Fig 7C–7E; two-way ANOVA for age and TLa ablation: significant main effect of ablation F[2,384] = 87.6, p < 0.001 and significant interaction effect F[8,384] = 13.0, p < 0.001; see Fig 7C for post-hoc tests). However, ablated fish were always larger than *kctd15* mutants (Fig 7C). Together, these results suggest that *kctd15* mutants are smaller in size at least in part due to the missing TLa, implicating the TLa in growth regulation in zebrafish.

## Discussion

In this work, we generated and characterized mutations in the zebrafish *kctd15* paralogs to better understand the role of Kctd15 in development. Homozygous mutants in either paralog alone showed no obvious phenotype or delay in growth or fertility, but *kctd15* double mutants, while viable and fertile, exhibit several phenotypes. These include defects in the NC domain and in NC derivatives, a growth delay and small size phenotype, and missing the torus lateralis, a region in the mid-brain. Among these phenotypes, the effect on the NC was expected on the basis of earlier work. Kctd15a/b morpholino injected embryos show an increase in NC markers and increased pigmentation, as also seen in the mutants. In addition the morphants show multiple morphological defects and larval lethality [6]; these defects are not seen in the mutants and thus are likely to be caused by non-specific effects of the morpholinos. Regarding the mechanistic basis of these phenotypes, we have shown that Kctd15 inhibits Wnt signaling and the activity of transcription factor AP-2 [6, 7]. These molecular effects are supported by the fact that Kctd1, a family member closely related to Kctd15, is likewise known to affect the Wnt and Ap-2 pathways [65, 66], and by the established requirement for these two pathways in NC formation and differentiation [31, 67–71]. We have suggested that Kctd15 functions to restrict the size of the NC domain. Its absence in the double *kctd15a/b* mutant likely leads to imbalance of NC cells rather than a whole-sale increase in the NC and its derivatives. This interpretation agrees with our observations that *kctd15* mutants show selective defects in NC derivatives such as increases in melanophores and xantophores but apparently not iridophores, and in the malformation in the craniofacial skeleton that is restricted to the maxillary segment. Thus, Kctd15 function is required for the normal regulation of just some but not all NC derivatives. Whether this is due to strength and restricted location of Kctd15 in wild type embryos or a specific effect on some but not other precursor cells is presently not known.

Beyond the expected effects on NC development, novel phenotypes were observed in the *kctd15* mutants. The generalized growth delay/small size phenotype may be secondary to the loss of the torus lateralis which is a site of GHRH production [63]. Moreover, the torus lateralis is immediately adjacent to the inferior lobe of the hypothalamus, which was recently implicated in visual control of feeding behavior [72]. The fact that TLa ablation only partially phenocopies the mutant growth delay may be due to contributions of other organs, incomplete ablation, or regeneration of the TLa in ablated wild type larvae. Finally, growth rates may also be affected by the loss of barbels which carry taste receptors [58, 73]; their absence may thus lead to reduced feeding. The loss of barbels may be due to the malformation of the maxillary bone (Fig 4F and 4G), and thus ultimately to the misregulation of the NC in *kctd15* mutants. The molecular mechanism that leads to loss of the torus lateralis in the mutants is not understood. The Wnt signaling pathway is involved in many developmental processes, and thus its misregulation in the *kctd15* mutants might be responsible for this phenotype. The AP-2 pathway is not quite as ubiquitously functioning in development as the Wnt pathway, yet AP-2 is known to interact with many proteins in the regulation of the expression of many genes (http://reactome.org/); however, no direct connection to midbrain development is apparent. In addition, it is possible that Kctd15 has molecular functions beyond the known effects on Wnt and Ap-2. Different members of the Kctd family have varied molecular functions, including as cofactors for E3 ubiquitin ligases and regulators of the hedgehog pathway [74, 75]. Thus, the range of molecular functions of Kctd15 may still not be fully explored, but the existence of mutations may help in future studies on this regulatory factor.

## Materials and methods

### Zebrafish maintenance and transgenic lines

Zebrafish (*Danio rerio*, AB strain) were maintained at 28.5°C [76] and embryos were staged according to [77] following all Animal Care Standard Operating Procedures. To prevent pigmentation in zebrafish larva, embryos were treated with 0.003% phenylthiourea (PTU) after 18 somites. *Tg(1*.*5kb kctd15a-GFP)* was generated through cloning the 1.5kb segment upstream of the *kctd15a* transcription start site upstream of a GFP reporter, and integrating this construct into the genome using *Tol2*.

### TALEN construction and injection

TALENs were designed using the TAL Effector Nucleotide Targeter (http://tale-nt.cac.cornell.edu) and assembled using the Golden Gate vector system [23], using modified pCS2TAL3 vectors [78]. Capped mRNAs were synthesized using the mMESSAGE mMACHINE SP6 kit (Ambion), checked on formaldehyde gels, and injected into 1-cell embryos, each at a concentration of 100 pg/nl. TALEN efficiency was checked by pooling 10 injected embryos at 48 hpf, PCR-amplifying a 150bp region around the expected cut site, cloning into pGEMT and sequencing several individual clones. Embryos were grown to adulthood and outcrossed to wild-type AB fish to identify founders and establish mutant lines carrying single mutations.

### Cell culture, protein expression and Western blotting

HEK293T cells were grown in Dulbecco’s modified Eagle’s medium supplemented with 10% FBS. Constructs containing the open reading frame of wild-type and mutant *kctd15a* and *kctd15b* sequences were cloned from 2-day cDNA of AB or homozygous mutant fish into pCS2+ or pCS2-Flag (N-terminal). These constructs were transfected into HEK293T cells using Xtreme-Gene HP (Roche) and treated according to [7]. Antibodies and dilutions used for Western Blot detection were: anti-Kctd15 (LSBio, 1:1000), anti-Flag (Sigma,1:5000), alpha-tubulin (Calbiochem, 1:1000); HRP-conjugated secondary antibodies were from Jackson (1:2000–1:5000).

### Embryo fixation and whole mount in situ hybridization

Embryos were fixed in 4% paraformaldehyde for 2 hr at room temperature or overnight at 4°C, dehydrated in a series of methanol washes (25%, 50%, 75%, 100%), and incubated at -20°C overnight before use. *foxd3*, *sox10*, and *dlx3b* primers were from [6]. All other probe templates were amplified from cDNA sequences with a reverse primer containing the T7 promoter sequence. Dig-labeled (Roche) probes were generated using the appropriate RNA polymerase (SP6 or T7, Roche) and standard protocols. *In situ* hybridization (ISH) was performed as in [6]. Probes were detected using BM Purple (Roche). Embryos were photographed on a Leica MZ16F with a Leica DFC500 camera. All primer sequences for probes are available upon request.

### qPCR and relative expression analysis

RNA was extracted from homogenized embryos and larva at the desired time using the TRIzol reagent and manufacturer’s protocol (Invitrogen). cDNA was synthesized using the QuantiTect Reverse Transcription Kit (Qiagen) and 25ng was used as a template. Primers were designed to amplify a region spanning an exon-exon border to avoid possible background from any genomic DNA contamination. *e1fa* was used as a control; all primer sequences are available upon request. qPCR was performed using SsoAdvanced Universal SYBR Green Supermix and protocol (Bio-Rad). All calculations for relative expression levels were done using the comparative C_T_ method described by [79]. All wild-type expression levels were normalized to *e1fa* expression and set to 1.0; mutant expression levels were calculated relative to wild-type.

### Alcian blue and Alizarin red stainings

For larval staining, cartilage (Alcian blue) and bone (Alizarin red) were stained simultaneously according to [56]. Adult craniofacial bones were stained using Alizarin red, followed by dissection of the anterior jaw elements and removal of remaining scales.

### Torus lateralis (TLa) brain scanning and sectioning

Larvae at 6 dpf were fixed in 4% PFA and labeled using an anti-tERK primary antibody (1:500, Cell Signaling, 4696) and Alexa488 secondary antibody. Confocal scanning, followed by brain registration and analysis was performed as reported [62]. To detect statistically significant changes in brain structure we scanned 14 wildtype and 14 mutant brains, co-registered them to the Brain Browser atlas [62], then performed t-tests between all individual pixels within the brain. To control for multiple comparisons, we set significance thresholds by repeating this procedure 1000 times, each time randomly assigning each brain to one of two groups. For each such permutation, we recorded the most significant t-test result. We then ranked all 1000 results, and used the 95th percentile t-test result as our significance threshold for the comparison between the actual wildtype and mutant groups, thus representing a 5% false positive rate. Finally we gaussian smoothed the map of significant pixels to produce the image in Fig 6C. The region missing in our mutants was identified by alignment with an adult reference brain [61]. Adult brains were dissected from 5 month old AB and *kctd15* mutant fish. Paraffin sectioning followed by H+E staining was performed on brain slices every 10 microns spanning the midbrain (HistoServ Inc, Germantown Maryland), in order to ensure that the presence or absence of the TLa could be ascertained.

### TLa ablation studies

*Tg(1*.*5kbup-kctd15a-GFP)* 5 dpf larvae were anesthetized and embedded in 2.5% LMP agarose/tricaine and the TLa bilaterally ablated using a 2-photon laser (Spectra-Physics MaiTai DeepSee). As a control, cells in the pallium region were bilaterally ablated in another group. After ablation, larvae were freed from agarose and allowed to recover in E3 medium for 24 hours before transfer to tanks for ad libitum feeding. Measurements were taken weekly for 5 weeks; total length was measured at 6 dpf and standard length (SL) was measured from 14 dpf onwards.

## Supporting information

S1 Fig Generation and confirmation of *kctd15* mutants. A) Mutations generated in the second exon of the *kctd15a* locus using TALENS (targeted DNA sequences in red). Examples of mutations discovered in the germ line of different founder fish are listed. For results presented in this paper, we used the 23 bp deletion, which resulted in a premature stop codon after 11 amino acids. B) Mutations generated in the third exon of the *kctd15b* locus using TALENS targeting DNA sequences shown in red (splice acceptor site in bold italic). Examples of mutations discovered in the germ line of different founder fish are listed. For results presented in this paper, we used the 19 bp deletion, which resulted in a premature stop codon in the middle of the BTB domain. C) Western blot of protein samples from cell extracts after induction of wildtype and mutant transcript expression. Antibodies recognizing an epitope at the C-terminal end of Kctd15, an N-terminal FLAG tag, and alpha-tubulin were used (see Methods). No proteins from either mutant transcript were detected. D) Quantitative PCR (qPCR) of *kctd15a* and *kctd15b* transcript levels in double mutant embryos showed transcript upregulation compared to wild-type.
(TIF)

S2 Fig Genes known to be involved in melanophore development that are unaffected by loss of Kctd15. Expression of *mitfa* is unchanged at 25 hpf (A-C), and only shows up-regulation at 48 hpf (C), after establishment of melanophore cells. A similar pattern is seen with *dct* transcripts at 25 and 48 hfp (D). Expression levels of *tyr* (E) or *kita* (F) are unaffected in our mutants compared to wild-type.
(TIF)

S3 Fig **There is no early up-regulation of mature xanthophore number in our mutants, as visualized by Pax7 antibody staining (A,B).** Other gene markers known to be involved in the specification of xanthophores early in development, including *csf1ra* (C), *xdh* (D) and *pax7b* (E) are not up-regulated.
(TIF)

S4 Fig Expression levels of genes known to be involved in iridophore specification, including *ednrba* (A), *ltk* (B), *pnp4a* (C) and *tfec* (D) were unchanged at 25 and 48 hpf in *kctd15* mutants compared to wildtype siblings.
(TIF)

S5 Fig Loss of Kctd15 has no effect on glial cell markers in early development. Expression of *foxd3* (A,B) and *sox10* (C,D) transcripts at 48 hpf in *kctd15* mutants shows no change in expression patterns of either cranial glia or trunk glia.
(TIF)

S6 Fig *kctd15* mutants lack all facial barbels. WT fish have 2 sets of facial barbels, nasal and maxillary (A), both of which are missing in *kctd15* mutants (B,C). While WT fish may have fewer than 4 due to a loss for several reasons, *kctd15* mutants never have any.
(TIF)

S7 Fig *Kctd15* mutants show *gh* RNA levels similar to wild type. *gh* levels were examined by qPCR (A) and *in situ* hybridization (B) in WT and mutant embryos at 48 hpf. While there is a general trend towards lower *gh* levels, this difference is not significant. In ~60% of embryos, the staining pattern of *gh* transcripts appears more sparse (in fewer cells), when compared to the rosette pattern observed in WT.
(TIF)

S8 Fig GFP expression in the TLa. Single horizontal (A) and coronal (B) z-stack images taken during confocal scanning of Tg(1.5kb-*kctd15a*-GFP) show GFP expression in the TLa. The heat maps in the single slices indicate the region missing in our *kctd15* mutants.
(TIF)
