# Supplementary figures and images for: Generation and characterization of Kctd15 mutations in zebrafish
Source: PLoS One. 2017 Dec 7;12(12):e0189162. doi: 10.1371/journal.pone.0189162 (PMC5720732; doi:10.1371/journal.pone.0189162)

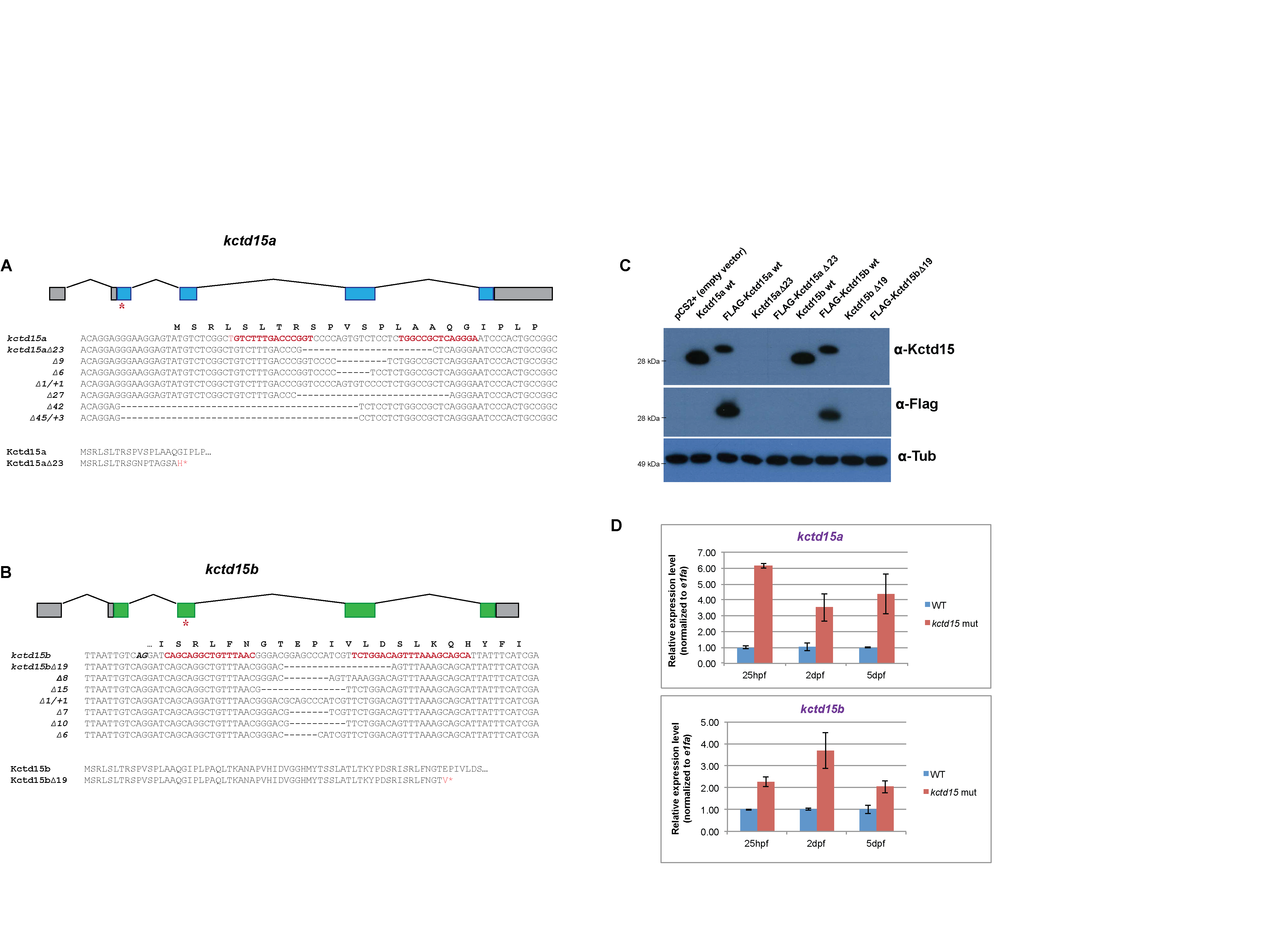

Supplement: S1 Fig — A) Mutations generated in the second exon of the kctd15a locus using TALENS (targeted DNA sequences in red). Examples of mutations discovered in the germ line of different founder fish are listed. For results presented in this paper, we used the 23 bp deletion, which resulted in a premature stop codon after 11 amino acids. B) Mutations generated in the third exon of the kctd15b locus using TALENS targeting DNA sequences shown in red (splice acceptor site in bold italic). Examples of mutations discovered in the germ line of different founder fish are listed. For results presented in this paper, we used the 19 bp deletion, which resulted in a premature stop codon in the middle of the BTB domain. C) Western blot of protein samples from cell extracts after induction of wildtype and mutant transcript expression. Antibodies recognizing an epitope at the C-terminal end of Kctd15, an N-terminal FLAG tag, and alpha-tubulin were used (see Methods). No proteins from either mutant transcript were detected. D) Quantitative PCR (qPCR) of kctd15a and kctd15b transcript levels in double mutant embryos showed transcript upregulation compared to wild-type. (TIF) [file pone.0189162.s001.tif]

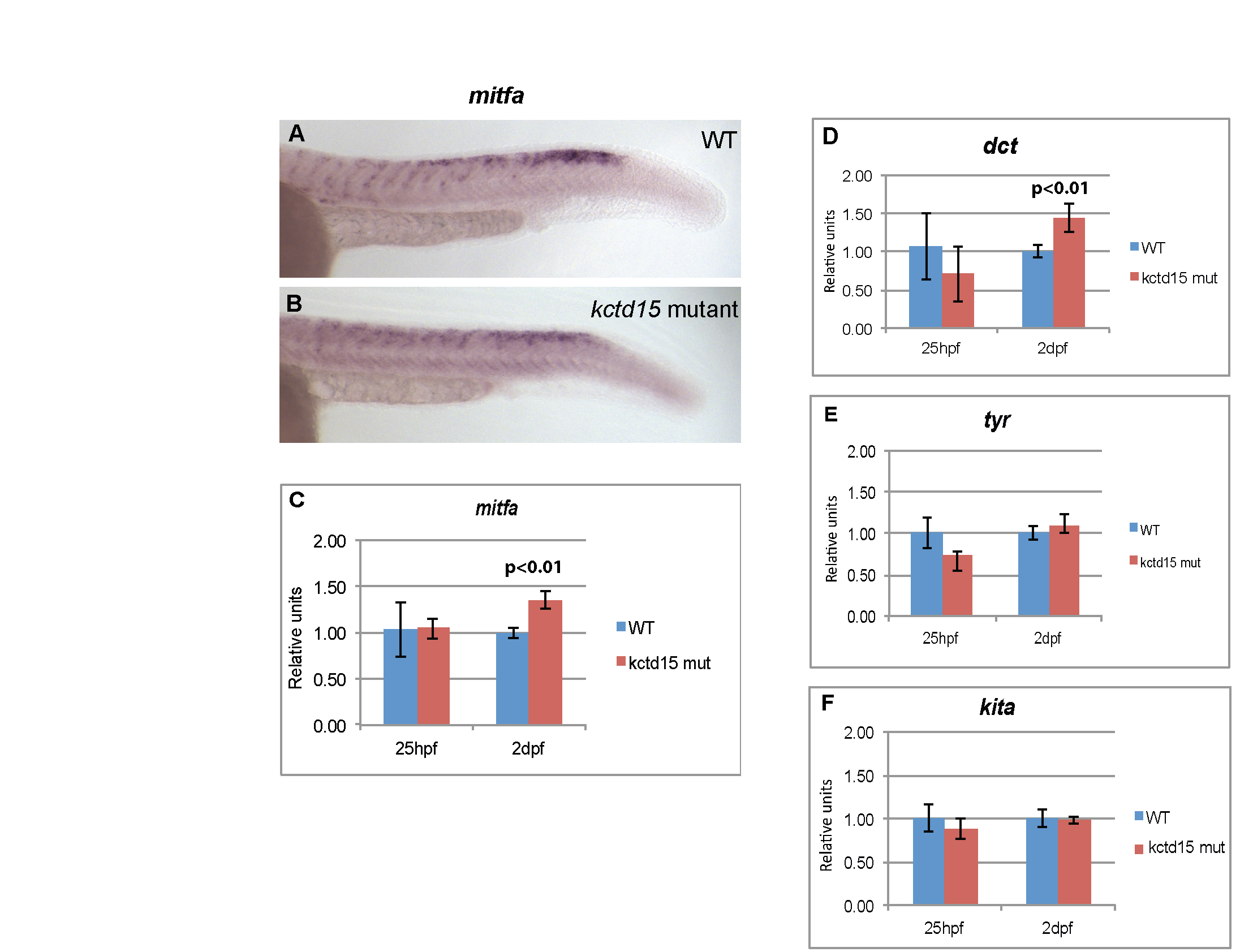

Supplement: S2 Fig — Expression of mitfa is unchanged at 25 hpf (A-C), and only shows up-regulation at 48 hpf (C), after establishment of melanophore cells. A similar pattern is seen with dct transcripts at 25 and 48 hfp (D). Expression levels of tyr (E) or kita (F) are unaffected in our mutants compared to wild-type. (TIF) [file pone.0189162.s002.tif]

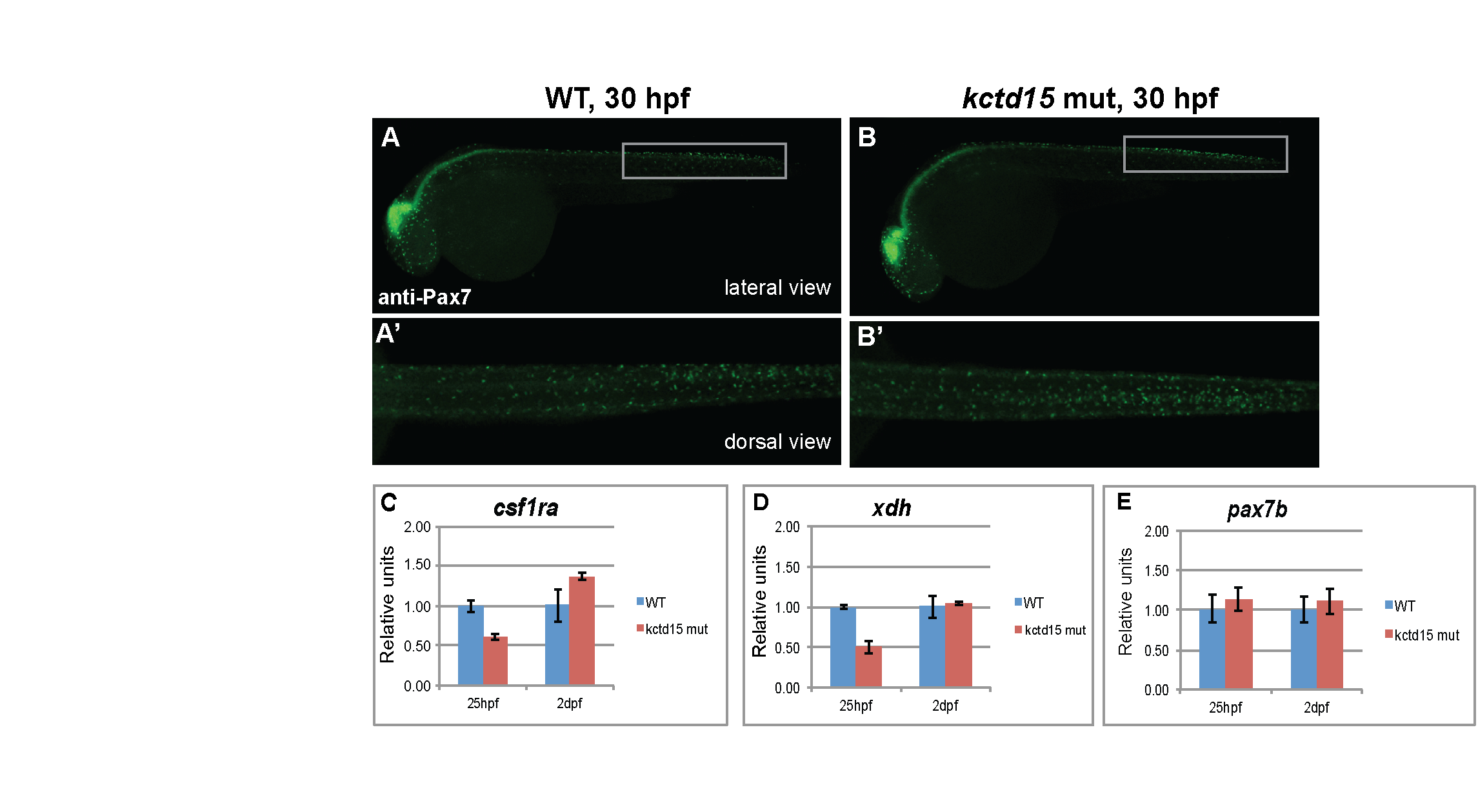

Supplement: S3 Fig — There is no early up-regulation of mature xanthophore number in our mutants, as visualized by Pax7 antibody staining (A,B). Other gene markers known to be involved in the specification of xanthophores early in development, including csf1ra (C), xdh (D) and pax7b (E) are not up-regulated. (TIF) [file pone.0189162.s003.tif]

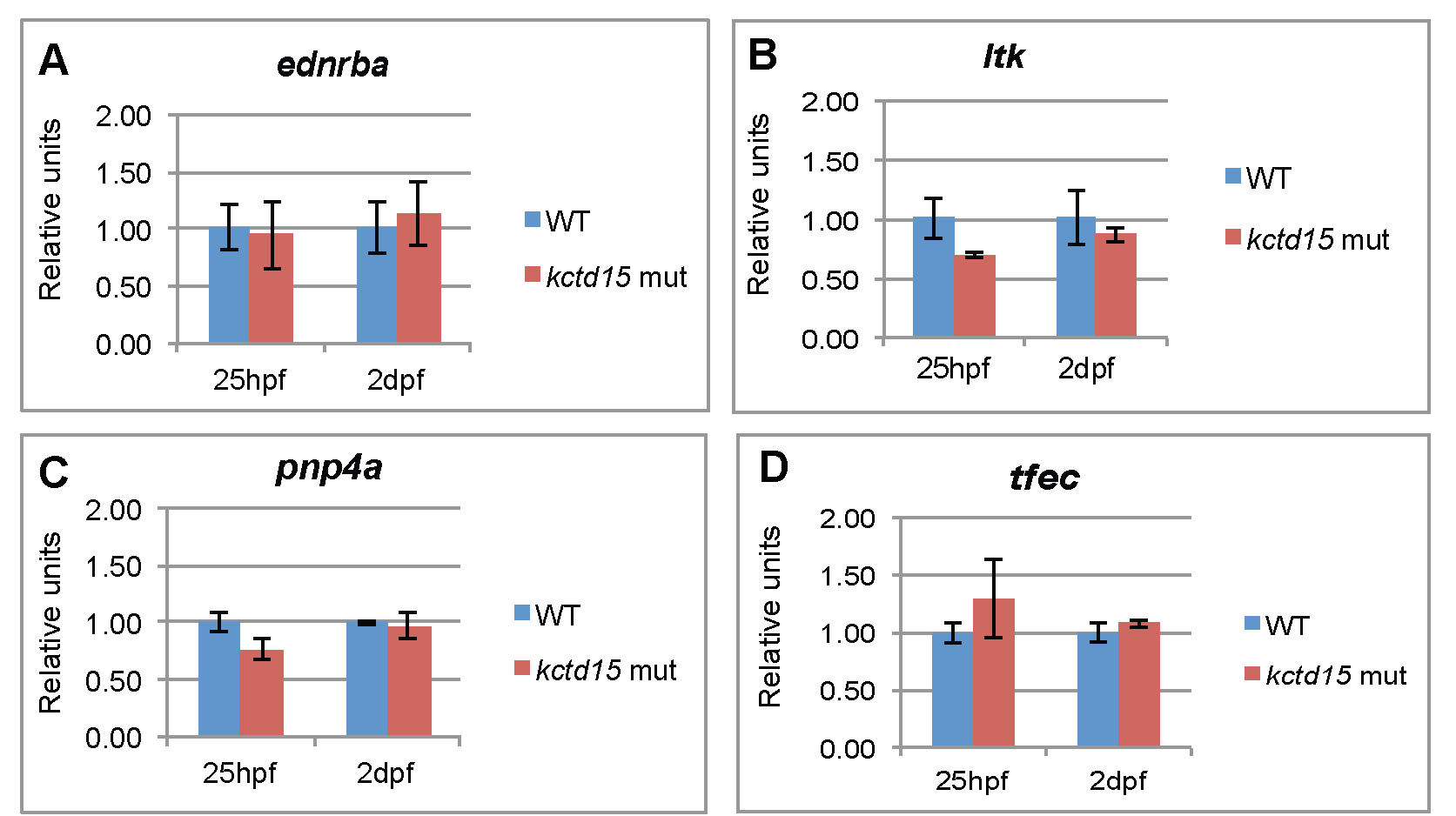

Supplement: S4 Fig — Expression levels of genes known to be involved in iridophore specification, including ednrba (A), ltk (B), pnp4a (C) and tfec (D) were unchanged at 25 and 48 hpf in kctd15 mutants compared to wildtype siblings. (TIF) [file pone.0189162.s004.tif]

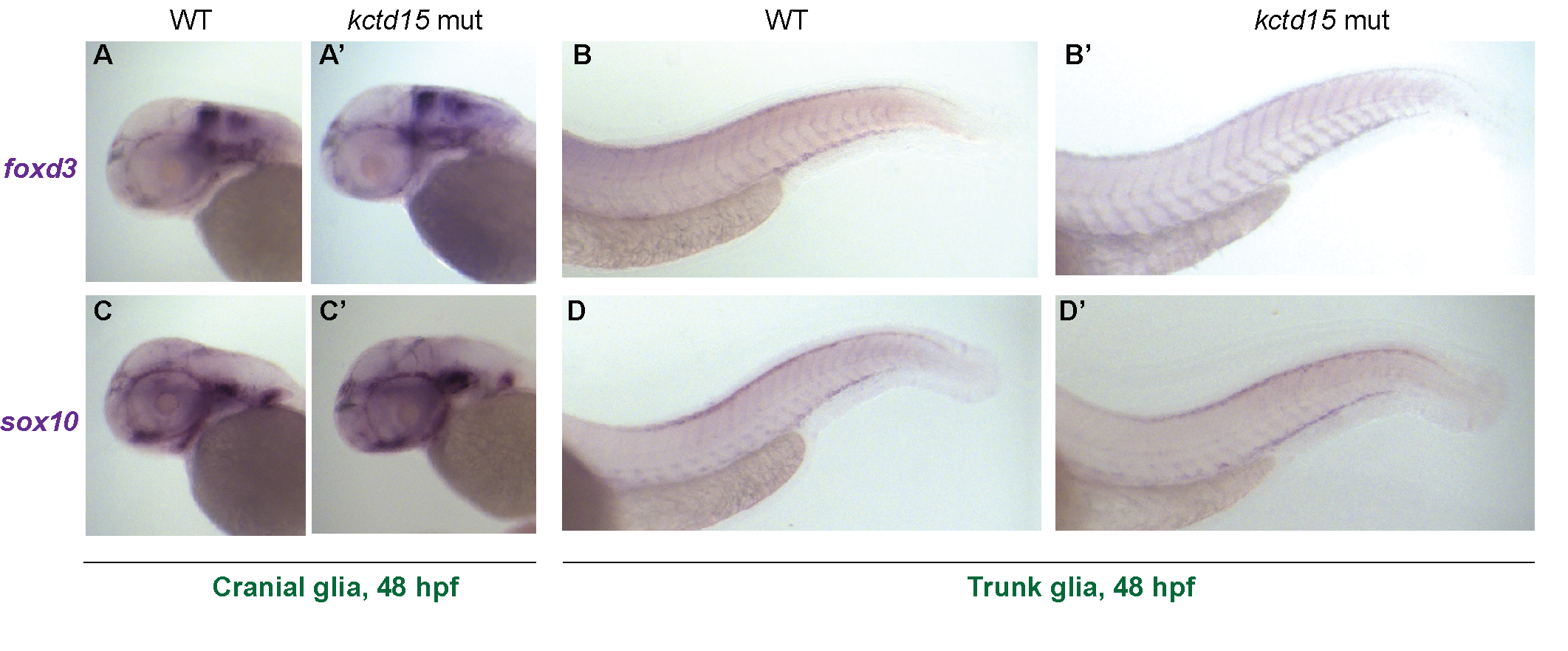

Supplement: S5 Fig — Expression of foxd3 (A,B) and sox10 (C,D) transcripts at 48 hpf in kctd15 mutants shows no change in expression patterns of either cranial glia or trunk glia. (TIF) [file pone.0189162.s005.tif]

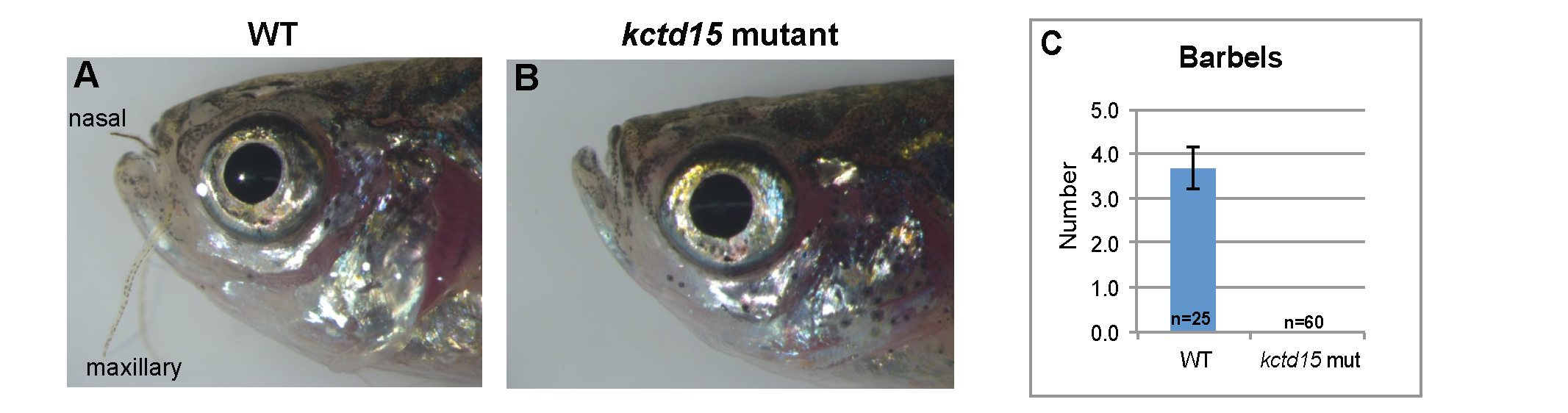

Supplement: S6 Fig — WT fish have 2 sets of facial barbels, nasal and maxillary (A), both of which are missing in kctd15 mutants (B,C). While WT fish may have fewer than 4 due to a loss for several reasons, kctd15 mutants never have any. (TIF) [file pone.0189162.s006.tif]

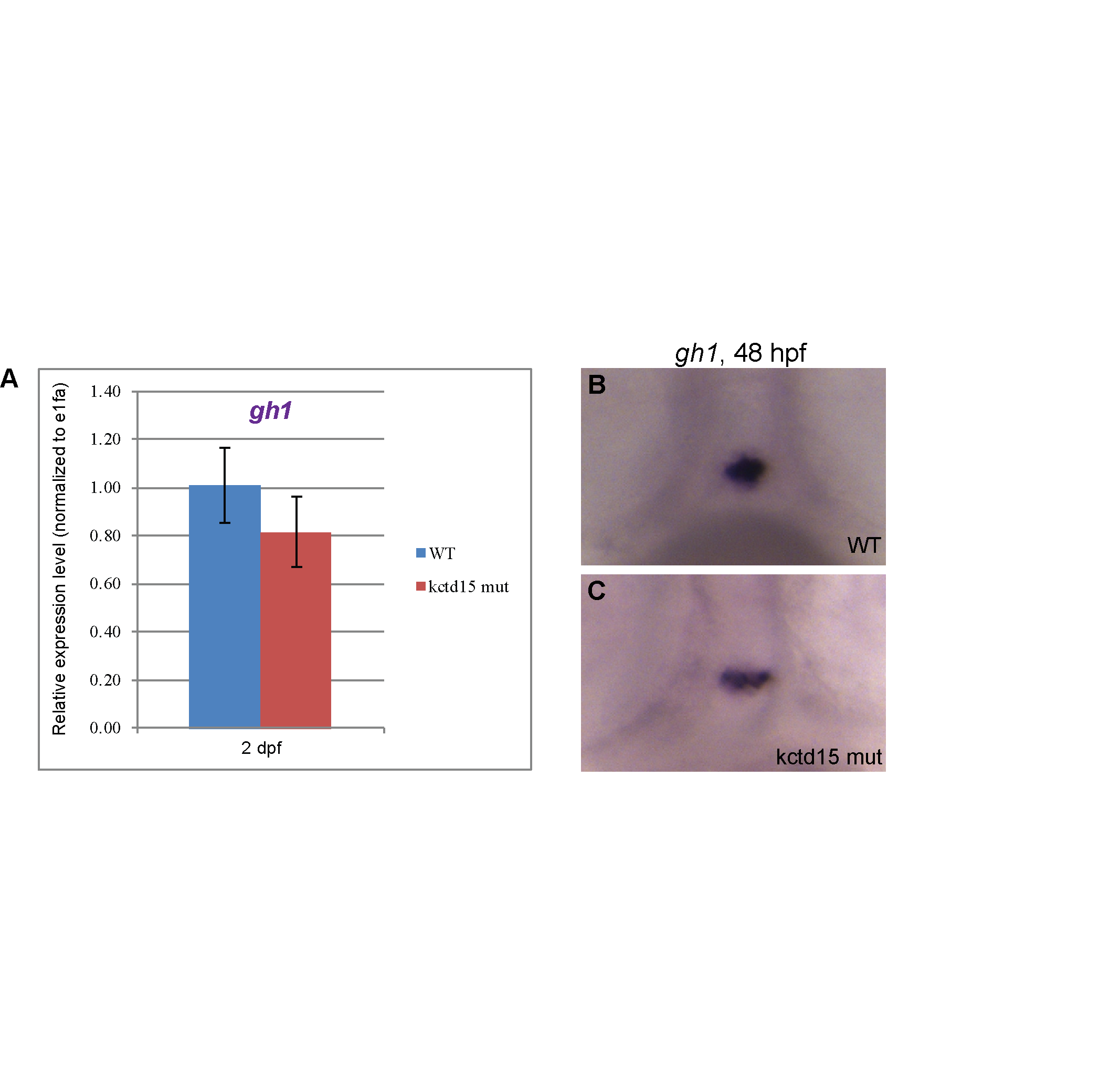

Supplement: S7 Fig — gh levels were examined by qPCR (A) and in situ hybridization (B) in WT and mutant embryos at 48 hpf. While there is a general trend towards lower gh levels, this difference is not significant. In ~60% of embryos, the staining pattern of gh transcripts appears more sparse (in fewer cells), when compared to the rosette pattern observed in WT. (TIF) [file pone.0189162.s007.tif]

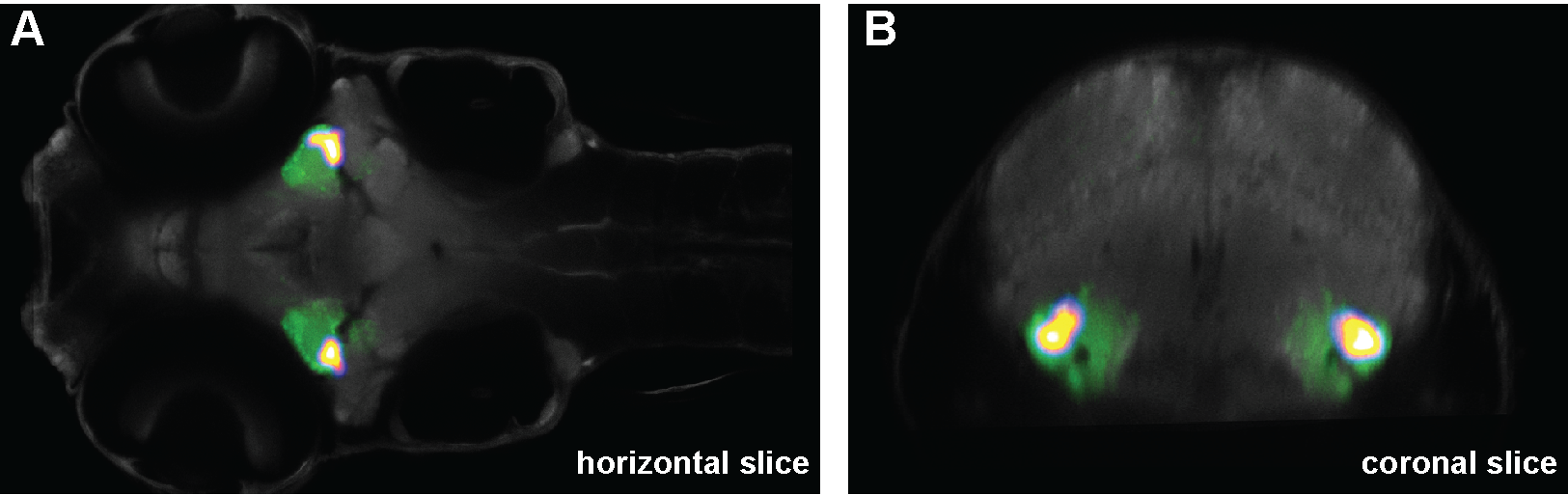

Supplement: S8 Fig — Single horizontal (A) and coronal (B) z-stack images taken during confocal scanning of Tg(1.5kb-kctd15a-GFP) show GFP expression in the TLa. The heat maps in the single slices indicate the region missing in our kctd15 mutants. (TIF) [file pone.0189162.s008.tif]
